# A cross-sectional examination of psychological distress, positive mental health and their predictors in medical students in their clinical clerkships

**DOI:** 10.1186/s12909-017-1035-8

**Published:** 2017-11-17

**Authors:** Inge van Dijk, Peter L. B. J. Lucassen, Chris van Weel, Anne E. M. Speckens

**Affiliations:** 1 Department of Psychiatry, Radboud University Medical Center, Nijmegen, the Netherlands; 2 Department of Primary and Community Care, Radboud University Medical Center, Nijmegen, the Netherlands; 3 Department of Health Services Research and Policy, Australian National University, Canberra, Australia

**Keywords:** Clinical clerkships, Medical students, Multiple linear regression, Positive mental health, Psychological distress

## Abstract

**Background:**

Medical students can experience the transition from theory to clinical clerkships as stressful. Scientific literature on the mental health of clinical clerkship students is scarce and mental health is usually defined as absence of psychological distress without assessing psychological, emotional and social wellbeing, together called ‘positive mental health’. This cross-sectional study examines the prevalence of psychological distress and positive mental health and explores possible predictors in a Dutch sample of clinical clerkship students.

**Methods:**

Fourth-year medical students in their first year of clinical clerkships were invited to complete an online questionnaire assessing demographics, psychological distress (Brief Symptom Inventory), positive mental health (Mental Health Continuum- SF), dysfunctional cognitions (Irrational Beliefs Inventory) and dispositional mindfulness skills (Five Facet Mindfulness Questionnaire). Multiple linear regression analysis was used to explore relationships between psychological distress, positive mental health (dependent variables) and demographics, dysfunctional cognitions and dispositional mindfulness skills (predictors).

**Results:**

Of 454 eligible students, 406 (89%) completed the assessment of whom 21% scored in the clinical range of psychological distress and 41% reported a flourishing mental health. These proportions partially overlap each other. Female students reported a significantly higher mean level of psychological distress than males. In the regression analysis the strongest predictors of psychological distress were ‘acting with awareness’ (negative) and ‘worrying’ (positive). Strongest predictors of positive mental health were ‘problem avoidance’ (negative) and ‘emotional irresponsibility’ (negative).

**Conclusions:**

The prevalence of psychopathology in our sample of Dutch clinical clerkship students is slightly higher than in the general population. Our results support conclusions of previous research that psychological distress and positive mental health are not two ends of one continuum but partially overlap. Although no conclusion on causality can be drawn, this study supports the idea that self-awareness and active, nonavoidant coping strategies are related to lower distress and higher positive mental health.

## Background

Clinical clerkships are a valuable part of medical education in which students can lay the foundation for their professional development. Unfortunately, the transition from theory to practice can result in problems related to professional socialization, high workload and heightened levels of psychological distress [1–5]. Although this transition is known to be stressful, scientific literature on the mental health of clinical clerkships students is scarce compared to literature on pre-clinical students and response rates are modest [6–9]. Prevalence rates of clinical clerkship students scoring above the cut-off for psychopathology vary from 27 to 48% [6, 8, 10, 11]. Mental health problems during clerkships are a predictor of postgraduate mental health problems in need of treatment [12, 13]. Although there are no data available on the relationship between student psychopathology and their actual performance during clinical clerkships, we do know that higher distress, burnout, and depression in residents are associated with more self-perceived errors [14–17].

Mental health as ‘presence or absence of disease’ is only one approach to the well-being of medical students. In 2011, Huber et al. opened a discussion with their paper ‘How should we define health’, by stating that we should move away from the static WHO definition of health as ‘a state of complete physical, mental and social well-being and not merely the absence of disease or infirmity’ [18]. They suggest to use the more dynamic definition ‘health is the ability to adapt and to self-manage’, because this reflects the capacity to cope, maintain and restore one’s integrity. In research on mental health, adopting this view could mean a shift from the emphasis on presence or absence of psychological distress to a broader definition in which also characteristics of psychological wellbeing and coping are used to determine a person’s mental health.

**Fig. 1:**
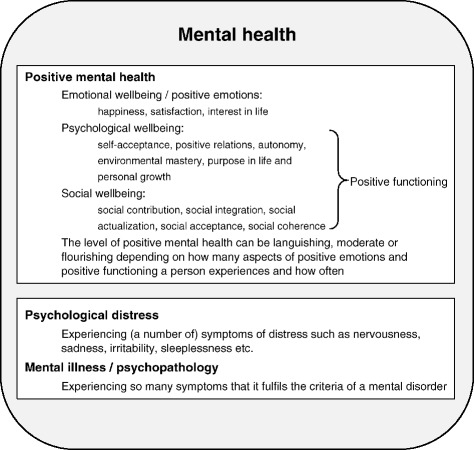
Overview of terminology as used in the current paper

Already for over a decade, Carol Ryff, professor of psychology at the University of Wisconsin, is studying psychological wellbeing of which she distinguishes six dimensions: self-acceptance, positive relations, autonomy, environmental mastery, purpose in life and personal growth [19]. Keyes and colleagues combined these dimensions of psychological wellbeing with dimensions of emotional wellbeing (e.g. being happy, interested in life and satisfied) and social wellbeing (e.g. feeling part of a community) which together he called ‘positive mental health’ or just ‘mental health’. In this paper, we will use ‘positive mental health’ as term for psychological, emotional and social wellbeing while ‘psychological distress’ represents the experience of symptoms of distress such as sadness, sleeplessness or anxiety. Positive mental health and psychological distress both fall within the scope of the overarching concept ‘mental health’ (see Fig. 1).

Positive mental health and psychological distress are not two ends of a single continuum, but two related yet distinctive concepts, which are complementary to each other and show some overlap [20, 21]. The absence of psychological distress can contribute to a positive mental health but does not guarantee or equal positive mental health and vice versa. For example, a person with a mental disorder such as an obsessive-compulsive disorder can at the same time feel happy, experience a purpose in life and have positive relations, which are aspects of positive mental health. The combination of levels of positive mental health and psychological distress is a better predictor of psychosocial functioning of a person than one of these alone [20, 22]. A cross-sectional study of Keyes and colleagues among 5689 college students showed that higher levels of positive mental health were associated with less suicidal behaviour and academic impairment in students both with and without a current mental illness [23]. Specifically in medical students, the prevalence of suicidal ideation, serious thoughts of dropping out and the prevalence of unprofessional behaviours declined as positive mental health improved [24].

Examining predictors of psychological distress and positive mental health could heighten understanding of how not only quality of life of students but indirectly also quality of patient care could be influenced positively. Looking at what is known about the predictors of psychological distress in medical students; neuroticism, perfectionism, high reality weakness, low maternal care, high performance-based self-esteem, disengagement and type A personality were found to be positively correlated with depressive symptoms [8, 13, 25]. While self-actualization, self-awareness and a sense of fulfilment showed an inverse correlation with depression [25]. Predictors of positive mental health (as defined by Ryff) in medical students have not been studied yet.

### Current study

With the current cross-sectional study we first aim to determine the prevalence of psychological distress and positive mental health in a sample of Dutch medical students in their clinical clerkships. Second, by means of regression analysis we will explore predictors of psychological distress and positive mental health from a number of demographic characteristics, dysfunctional cognitions, and dispositional mindfulness skills. We chose to explore the demographic characteristics gender, marital status, religion and nationality as in previous research in other target groups all of them were associated with levels of psychological distress and/or psychological wellbeing [26–35].

The dysfunctional cognitions we are interested in (‘worrying’, ‘rigidity, ‘need for approval, ‘emotional irresponsibility’ and ‘problem avoidance’) are based on Ellis’ theory on irrational cognitions people can have about themselves, for example, the idea that suffering and misfortune is beyond their influence (emotional irresponsibility) or that one needs other people’s approval to be happy (need for approval). We hypothesize the dysfunctional cognition ‘worrying’ to be a strong predictor of psychological distress as worrying is highly correlated to neuroticism, a known risk factor for psychological distress in the general population as well as in medical students [12, 13, 36, 37]. We expect a less strong connection of worrying to positive mental health.

We are also interested in dispositional mindfulness; the natural ability to be aware of one’s current thoughts, feelings and other experiences in a curious, non-judging way without being trained to do so. In other studies, higher levels of dispositional mindfulness were associated with lower levels of distress [38–41] and higher positive states of mind [42]. It also moderated the relationship between self-care and psychological distress and the relationship between self-control and psychological symptoms [40] and explained significant variance in psychological health [40].

## Methods

### Setting

The medical school curriculum of the Radboud University Medical Center in Nijmegen consists of a three-year pre-clinical Bachelor study in which students study medical theory followed by a three-year Master study consisting of clinical rotations in two- or three-month periods alternated by a one-month period of reflection, study and preparation for the next clerkship.

### Subjects

From February 2011 to August 2012 we invited all first-year clinical clerkship students in the fifth month of their clerkships to complete an online survey, which was accessible at home with a personalized link. All participating students gave informed consent before completing the survey.

### Ethical considerations

The study was embedded in two studies which were approved of by the medical ethical research committee Arnhem-Nijmegen.

### Outcome measures

#### Psychological distress

The Brief Symptom Inventory (BSI) is a 53-item questionnaire, measuring psychological symptoms of distress. It can be used in both clinical and non-clinical populations. It focusses on dimensions of somatization, cognitive problems, interpersonal sensitivity, depression, anxiety, hostility, phobic fear, paranoid thoughts and psychoticism [43]. A five-point Likert scale is used to score items from ‘*none-at-all’* to ‘*extremely’*. The mean score on all 53 items, which is called the ‘global severity index’ (range 0–4), is widely used in studies as a measure of overall psychological distress. A higher score implies a higher level of psychological distress. The Dutch BSI has been found to have a high reliability and high validity [44, 45]. A cut-off score of 0.58 or higher on the global severity index is proposed for screening on psychopathology [44].

#### Positive mental health

We used the 14-item Mental Health Continuum-Short Form (MHC-SF) as a measure of positive mental health, consisting of the three dimensions emotional, psychological and social wellbeing. Emotional wellbeing exists of three items assessing how often during the past month a person was feeling happy, interested in life and satisfied. Psychological wellbeing is measured by six items based on the model of Ryff assessing self-acceptance, positive relations, autonomy, environmental mastery, purpose in life and personal growth. Social wellbeing is assessed by five items asking about social contribution, social integration, social actualization, social acceptance and social coherence. Together the eleven items of social wellbeing and psychological wellbeing are called the items of positive functioning (see Fig. 1) [21]. All 14 items range from ‘*never’* to ‘*daily’* on a six-point Likert scale, total score range 0–70. A higher score indicates a higher level of positive mental health. The degree of positive mental health of a person can also be expressed in the categories ‘flourishing’, ‘moderate’ and ‘languishing’ mental health. Experiencing ‘every day’ or ‘almost every day’ at least one of the three items of emotional wellbeing and at least six of the eleven items of positive functioning during the past month or 2 weeks is considered ‘flourishing mental health’. ‘Languishing mental health’ is categorized in persons with a combination of low scores on at least one item of emotional well–being and on at least six items of positive functioning. All other combinations represent ‘moderate mental health’. Studied in a representative sample of the Dutch population, internal reliability was high (α = 0.89) and validity good for the Dutch total MHC-SF [21].

### Possible predictors

#### Demographic variables

We assessed age, gender, relationship status and religious background.

#### Dysfunctional cognitions

We used the five subscales of the 50-item Irrational Beliefs Inventory to assess students’ irrational cognitions, which are considered to be related to a person’s vulnerability for developing psychopathology [46]. The IBI is derived from the Irrational Beliefs Test [47] and the Rational Behaviour Inventory [48] but with improved psychometric quality. The subscales are worrying, rigidity, need for approval, problem avoidance, and emotional irresponsibility. They are rated on a five-point Likert scale from ‘*strongly disagree’* to ‘*strongly agree’*, total score range 50–250. A higher score indicates a higher level of dysfunctional cognitions. In a randomly selected Dutch university student sample validity and reliability (α = 0.83–0.85) were satisfactory [49, 50].

#### Mindfulness skills

With the 39-item Five Facet Mindfulness Questionnaire (FFMQ) five domains of mindfulness skills are assessed: observing, describing, acting with awareness, non-judging of inner experience and non-reactivity to inner experience [51]. Items are rated on a five-point Likert-type scale from *‘never or very rarely true’* to ‘*very often or always true’*, total score range 39–195. A higher score implying a higher level of mindfulness skills. The subscales of the Dutch FFMQ have been shown to have good internal consistency [52].

### Statistical analysis

We collected all data by means of an online survey tool (Limesurvey) and exported them to IBM SPSS statistics 21.0 for analysis.

#### Descriptive statistics

We used descriptive statistics to assess all variables. We used independent sample t-tests to compare age and gender of all participating students and non-participants and to compare positive mental health scores of men and women. For the dependent variable psychological distress, we used a non-parametric test to compare scores across men and women, because of skewed data. Despite this, we reported mean instead of median scores, for the sake of comparability across literature and clarity in reporting cut-off scores to determine individual cases.

#### Analyses

In preparation of performing multiple linear regression analyses, we used Kolmogorov-Smirnov tests and visual inspection (Q-Q plots, histograms) to assess normality of all variables. Assumptions of linearity, heteroscedasticity, and independence of residuals were assessed by means of inspecting residual plots. We assessed correlations between each of the two dependent variables and the independent variables (demographic characteristics, dysfunctional cognitions, and mindfulness skills) using Pearson’s correlation coefficient (*r)* and Spearman’s rho in case of non-normal distributions. Psychological distress and positive mental health were our primary outcome measures (dependent variables). We used the global severity index of the Brief Symptom Inventory as a measure of psychological distress and the total score of the Mental Health Continuum-SF as a measure of positive mental health. We performed multiple linear regression analyses to assess relationships between our dependent and independent variables. As students were grouped in classes at the time of inclusion, data was nested, therefore the intra-class correlation coefficient (ICC) was calculated for both dependent variables to assess if multilevel regression was necessary. In both dependent variables the ICC was redundant and therefore required no further multilevel analyses. Independent variables with significant correlations with the dependent variable were entered into the model simultaneously. Multicollinearity was assessed by means of correlation matrices, tolerance and Variance Inflation Factor (VIF) values. Durbin-Watson statistics were calculated to assess the dependence of variables. To investigate the unique relationship of psychological distress with the independent variables we corrected for positive mental health by entering it in the model. Also, to investigate the unique relationship of positive mental health with the independent variables, we entered psychological distress in the model. By means of backward elimination, variables with non-significant contributions to the model were removed. Both final models contained only the variables contributing significantly to the model. Squared semi-partial correlations were computed to determine the proportion of unique variance that each predictor contributed to the total explained variance of the model.

## Results

### Demographic characteristics

In total, 406 (89%) of 467 eligible students completed the online assessment. Of the 61 students refusing to participate, 48 provided data on gender and age, revealing no significant differences between participants and non-participants.

Table 1 shows that the vast majority of the students was female (75%) and in a relationship (58%). The percentage of 3% students being of non-Dutch nationality is exactly equal to regional statistics reported by Statistics Netherlands. About half of the students considered themselves religious, mainly Catholic. Missing values analysis showed that 6% of the cases contained missing values and that 2.4% of data were missing.

**Table 1:** Characteristics of participating clinical clerkship students (*n* = 406)

Demographic characteristics	
Age, median	23.0
Female gender, n (%)	306 (75.4)
Marital status, n (%)	
Single	170 (41.9)
In relationship, not married	225 (55.5)
Married	10 (2.5)
Divorced	1 (0.2)
Nationality, n (%)	
Dutch	388 (95.6)
German	9 (2.2)
Other	4 (0.9)
Missing	5 (1.2)
Religion, n (%)	
Atheist	196 (48.3)
Catholic	120 (49.6)
Protestant	24 (5.9)
Muslim	11 (2.7)
Other	49 (12.1)
Missing	6 (1.5)
Personal characteristics	
Dysfunctional cognitions, mean score (SD)	
Worrying (range 12–60)	33.5 (7.6)
Rigidity (range 14–70)	36.0 (5.8)
Need for approval (range 7–35)	23.5 (4.3)
Emotional irresponsibility (range 7–35)	22.2 (4.0)
Problem avoidance (range 10–50)	23.1 (4.9)
Mindfulness skills, mean score (SD)	
Observing (range 8–40)	21.6 (5.6)
Describing (range 8–40)	28.0 (5.9)
Acting with awareness (range 8–40)	29.7 (5.6)
Non-judging (range 8–40)	30.9 (6.0)

### Prevalence of psychological distress and positive mental health

Overall, 21% of the students reported a level of psychological distress above the cut-off score for psychopathology (Table 2). Female students reported a significantly higher mean level of psychological distress than male students. A flourishing mental health was reported by 37.5% of the female students, and 51% of the male students, a non-significant difference after correction for multiple testing. Table 3 shows that psychological distress and positive mental health are not two ends of one continuum but partially overlap. The majority of students with high levels of distress (77%) report a moderate positive mental health and 18% of them reports a flourishing positive mental health.

**Table 2:** Psychological distress and positive mental health in participating clinical clerkship students

	Total	Women	Men	*p* value^b^
Psychological distress				
Global severity index, M (SD)	0.39 (0.29)	0.41 (0.29)	0.30 (0.28)	<0.001^c^
Above cut-off^a^, n (%)	86 (21.2)	72 (23.6)	14 (14.0)	.05
Positive mental health				
Total score, M (SD)	45.7 (10.3)	45.3 (10.1)	46.8 (10.8)	.21
Positive mental health, categories				
Languishing, n (%)	5 (1.3)	4 (1.4)	1 (1.0)	.07
Moderate, n (%)	225 (57.8)	179 (61.1)	46 (47.9)	
Flourishing, n (%)	159 (40.9)	110 (37.5)	49 (51.0)	

^*a*^ *a cut-off score of 0.58 is advised in Dutch samples to screen for psychopathology*

^*b*^ *In case of skewed/categorised data non-parametric Mann-Whitney U tests and Chi-square tests were used*

^*c*^ *remained significant after Bonferroni correction for multiple testing 0.05/4 = alpha level 0.0125*

**Table 3:** Positive mental health in clinical clerkship students with low and high psychological distress

	Low distress^a^ (*n* = 307)	High distress (*n* = 82)
Positive mental health, categories		
Languishing mental health, n (%)	1 (0.3)	4 (4.9)
Moderate mental health, n (%)	162 (52.8)	63 (76.8)
Flourishing mental health, n (%)	144 (46.9)	15 (18.3)

^*a*^ *the advised score of 0.58 to screen for psychopathology was used as cut-off to distinguish between low and high distress*

### Multiple linear regression model of psychological distress

Correlations between psychological distress and the possible predictors are shown in Table 4. The final model for predictors of psychological distress is shown in Table 5. It contains the three significant predictors that remained after backward elimination of non-significant variables from the model. The mindfulness skill ‘acting with awareness’ was the strongest inversely correlated predictor for psychological distress, uniquely explaining 7.1% of total variance. The dysfunctional cognition ‘worrying’ uniquely explained 6.1% of the variance, making it the strongest positively correlating predictor. The mindfulness skill ‘non-judging’ made a smaller, but significant contribution uniquely explaining 2.6% of the variance. The final model explained 50% of the total variance in psychological distress.

**Table 4:** Correlations between psychological distress, positive mental health and possible predictors (*n* = 389)

	PD^a^	PMH
Demographic variables		
Gender (f/m)	−.23^**^	.06
Age (yrs)	−.06	−.03
Relationship (n/y)	−.12^*^	.05
Religious (n/y)	−.01	.07
Nationality (Dutch/other)	.01	−.03
Dysfunctional cognitions		
Worrying	.59^**^	−.35^**^
Rigidity	.00	-,02
Need for approval	.39^**^	−.19^**^
Emotional irresponsibility	.06	−.14^**^
Problem avoidance	.26^**^	−.29^**^
Mindfulness skills		
Observing	.16**	−.03
Describing	−.23^**^	.21**
Acting w. awareness	−.52^**^	.32**
Non-judging	−.51^**^	.29**
Non-reacting	−.19^**^	.19**

*PD* Psychological distress*, PMH* Positive mental health

* P < .05 ** P < .01

^*a*^ *spearman’s rho*

**Table 5:** Multiple linear regression model of psychological distress

	Psychological distress			
Predictors	Standardized β	t	Unique variance (%)	*P* value
Acting w. awareness	−0.31	−7.4	7.1	<.001
Worrying	0.30	6.8	6.1	<.001
Non-judging	−0.20	−4.5	2.6	<.001
Corrected for:				
Positive mental health	−0.14	−3.4	1.5	<.001
	Total model			
	*R* ^*2*^	*Adj. R* ^*2*^	*F*	*P*
	50.0	49.4	95.8	<.001

**Table 6:** Multiple linear regression model of positive mental health

	Positive mental health			
Predictors	Standardized β	t	Unique variance (%)	*P* value
Problem avoidance	−0.19	−3.9	3.1	<.001
Emotional Irresponsibility	−0.13	−2.8	1.6	<.01
Worrying	−0.12	−2.1	0.9	.037
Corrected for:				
Psychological distress	−0.27	−4.8	4.7	<.001
	Total model			
	*R* ^*2*^	*Adj. R* ^*2*^	*F*	*P*
	21.9	21.1	26.9	<.001

### Multiple linear regression model of positive mental health

Table 4 shows correlations between positive mental health and its possible predictors. The final model for predictors of positive mental health is shown in Table 6. It also contains three significant predictors after backward elimination and explains 21.9% of the variance in positive mental health. The dysfunctional cognition ‘problem avoidance’ is the strongest inversely correlated predictor explaining 3.1% of the variance, followed by ‘emotional irresponsibility’ explaining 1.6% of the variance and ‘worrying’ explaining 0.9% of the variance.

The highest Variance Inflation Factor score of our variables was 1.5 in the model of psychological distress and 1.6 in the model of positive mental health. In both models this value was well below 10 indicating that multicollinearity was not a problem.

## Discussion

In this study, 21% of students scored above cut-off level for psychopathology and 41% reported a flourishing mental health The inversely related mindfulness skill ‘acting with awareness’ was the strongest predictor for psychological distress and for positive mental health the strongest predictor was the dysfunctional cognition ‘problem avoidance’, also inversely related.

### Prevalence of psychological distress and positive mental health

The 21% of clinical clerkship students scoring above the cut-off for psychopathology is slightly lower than the 27% of Swedish students entering clinical training (Mini International Neuropsychiatric Interview) [8] and the 25% of Australian students in their internships (General Health Questionnaire-28) [11] and substantially lower than the 37% in Iranian clerkship students (General Health Questionnaire-28) [10].

It is even less than half the amount of the 48% as reported in the only other Dutch study by Gaspersz and colleagues, who screened for common mental disorders in a sample of pre-clinical and clinical students with combined response rate of 52%. This finding is in line with the conclusion of Hope et al. in their systematic review on depression, anxiety and psychological distress outside North-America, that studies with higher quality and a response rate higher than 80%, in general, find lower rates of psychological distress.

Regarding differences between countries; working environment and responsibilities of students during their clinical clerkships might vary. Also, the threshold that students experience to report symptoms of psychological distress could be influenced by cultural differences. Specifically for American students, one could hypothesize that the high study debt compared to other (European) countries might contribute to the difference in rates of psychopathology. However, scientific literature so far does not show a clear relationship between student level of debt and psychological distress [53] and between debt and positive mental health [24]. Debt was, however, significantly associated with suicidal ideation in the previous year, but not with future suicidal ideation [54].

The percentage of students reporting a flourishing mental health in our sample was twice as high as the percentage of students scoring above cut-off for psychopathology. Dyrbye et al. suggest a flourishing mental health can attenuate consequences of high levels of distress [24]. Students with a mental disorder but with flourishing positive mental health might be less at risk than those with languishing positive mental health. Investigating this further would give more information on which students might be in need of support.

### Predictors of psychological distress

The only other study examining predictors of psychological distress specifically in clinical clerkship students is a longitudinal Swedish study which explored effects of personality traits and study environment on psychiatric morbidity. Only depressive symptoms at first year remained a significant predictor of psychiatric morbidity at third year when entered in a model with the other potential predictors workload, worries about future competence, financial worries, impulsivity, negative affectivity, performance based self-esteem and disengagement at first year [8]. The finding that external factors such as workload and financial worries did not significantly predict psychopathology was also reported in studies among medical students from different years: A 1-year prospective longitudinal study in medical students from all 6 years in two Dutch medical faculties showed that mental health problems were not significantly predicted by exogenous study factors such as study delay, study hours per week, study stressors or having a part-time job but by the personal factors ‘worry about health’ (risk factor) and ‘excessive drinking behaviour’ (protective factor) [55]. This is similar to the results of Bore and colleagues, [56] who examined 20 possible endogenous and exogenous predictors of psychological distress among 127 Australian medical students from year 1 to 5. They found that not gender, demographics, hours studying, paid work or volunteer work were most important, but that emotional resilience was the strongest predictor of psychological distress. Emotional resilience could be described as being emotionally stable, calm and grounded as opposed to emotionally reactive (neurotic). This is in line with our findings that the endogenous factors acting with awareness (negative) and worrying (positive) are stronger predictors of psychological distress than demographic variables.

The only other study, besides ours, examining the relationship between dispositional mindfulness and psychological distress is that of Slonim and colleagues in Australian medical students from year 1 to 5. [38] Similar to our results, they also found the subscales ‘acting with awareness’ and ‘non-judging’ to be most strongly associated with lower levels of depression and anxiety. It is interesting that also in other target groups like for example men with advanced prostate cancer [57], fibromyalgia patients [58], and a community sample comprising of non-meditators and experienced meditators [59] of all FFMQ subscales ‘acting with awareness’ and ‘non-judging’ are strongest associated with lower psychological distress.

‘Acting with awareness’ refers to a person’s ability to focus on present moment experiences. Possibly, a person with a high level of self-awareness is less bothered by ruminative thoughts and more responsive to his own needs, which could decrease psychological distress. It could also be the other way around; a person experiencing a high level of psychological distress might be unable to focus on the present moment experience, therefore being less self-aware.

### Predictors of positive mental health

So far, no other studies have examined predictors of positive mental health in clinical clerkship students or other medical students. However, there are two Dutch studies in other target groups which have examined predictors of positive mental health. In a sample of 1161 Dutch participants from a representative internet panel between the ages of 18 and 88, agreeableness and extraversion were uniquely related to positive mental health whereas emotional instability was uniquely related to psychological distress. Agreeableness (being warm, empathic and friendly) and extraversion (being outgoing, social) are both aspects which are important in interpersonal relationships, an important aspect of positive mental health. The second Dutch study was a randomized controlled trial (*n* = 93) which showed that Acceptance and Commitment Therapy improved positive mental health of participants and that psychological flexibility during the intervention mediated the effects. That psychological flexibility, the ability to accept aversive internal experiences, is related to positive mental health seems similar to our finding that ‘problem avoidance’ and ‘emotional irresponsibility’ are its inversely related predictors. Coping with problems in an active way and maintaining an internal locus of control seem to improve positive mental health. Again this could also be the other way around; students with high positive mental health might be better able to approach problems in an active way and remain in control.

Above findings are in line with the definition of health as ‘the ability to adapt and to self-manage’. An approaching coping style can be seen as a healthy way to adapt to a new situation and self-management relates to the locus of control of students. Clinical clerkships are eminently a period in which students are confronted with complex situations, insecurity, and suffering, which requires the ability to adapt and self-manage to remain a healthy professional. Furthermore, the fact that psychological distress and positive mental health share the predictor ‘worrying’, but also have a few separate predictors is supportive for the hypothesis that they are related, but distinct concepts and not two ends of the same continuum.

### Strengths and limitations

Although it is also reported in other studies that the explained variance by personality traits is higher in psychopathology than in positive mental health [60], the fact that only 21% of variance in positive mental health was explained by our predictors compared to 50% of variance in psychological distress could mean that we did not manage to find the most important predictors of positive mental health yet. In light of the definition of Huber and colleagues it would be interesting to investigate the relationship between positive mental health and resilience, which is defined as ‘positive adaptation despite experiences of significant adversity or trauma.’ This might be more relevant to clinical clerkships than the dysfunctional cognitions that we used in our current study. Our study is cross-sectional in nature, therefore we cannot make any assumptions on causality. Also, we performed our study in one university medical centre, which could limit the generalizability of results. Strengths of our study are the high response rate (89%) and medium-large sample size.

## Conclusion

The prevalence of psychopathology in our sample of Dutch clinical clerkship students is slightly higher than in the general population. Although no conclusion on causality can be drawn, this study supports the idea that self-awareness and active, nonavoidant coping strategies are related to lower distress and higher positive mental health. Supporting the development of these skills might contribute to student wellbeing. The combination of levels of psychological distress and positive mental health might also be a better indicator of which students are in need of support than levels of psychological distress alone. More longitudinal research needs to be done to investigate these hypotheses.

## Abbreviations

BSI: Brief symptom inventory
FFMQ: Five facet mindfulness questionnaire
IBI: Irrational beliefs inventory
IBM: International business machines
ICC: Intra-class correlation coefficient
MHC-SF: Mental health continuum-short form
SPSS: Statistical package for the social sciences
VIF: Variance inflation factor
WHO: World Health Organization
